# Deep Sequencing Reveals the Complete Genome Sequence of *Sweet potato virus G* from East Timor

**DOI:** 10.1128/genomeA.00957-16

**Published:** 2016-09-08

**Authors:** Solomon Maina, Owain R. Edwards, Martin J. Barbetti, Luis de Almeida, Abel Ximenes, Roger A. C. Jones

**Affiliations:** a School of Plant Biology and Institute of Agriculture, Faculty of Science, University of Western Australia, Crawley, Western Australia, Australia; b Cooperative Research Centre for Plant Biosecurity, Canberra, Australian Capital Territory, Australia; c CSIRO Entomology, Floreat Park, Western Australia, Australia; d Seeds of Life Project, Ministry of Agriculture and Fisheries, Dili, East Timor; e DNQB-Plant Quarantine, Presidente Nicolau Lobato International Airport, Dili, East Timor; f Institute of Agriculture, Faculty of Science, University of Western Australia, Crawley, Western Australia, Australia; g Department of Agriculture and Food Western Australia, South Perth, Western Australia, Australia

## Abstract

We present the first complete *Sweet potato virus G* (SPVG) genome from sweet potato in East Timor and compare it with seven complete SPVG genomes from South Korea (three), Taiwan (two), Argentina (one), and the United States (one). It most resembles the genomes from the United States and South Korea.

## GENOME ANNOUNCEMENT

To examine possible connectivity between viruses infecting crops in northern Australia and Southeast Asia, we studied sweet potato viruses from East Timor and Australia. *Sweet potato virus G* (SPVG) is a single-stranded RNA virus within the genus *Potyvirus*, family *Potyviridae* (1). It has not been reported from East Timor or Australia. Seven complete SPVG genomes are available on GenBank: three from South Korea, two from Taiwan, and one each from Argentina and the United States (2, 3). East Timorese sample 66AL, collected in May 2015 from the Aileu district of East Timor, was sequenced and a complete SPVG genome was obtained.

Fifteen East Timorese samples blotted onto Fast Technology for Analysis of nucleic acids (FTA) cards (4) were sent to Australia. Total RNA was extracted from the FTA cards using the ZR Plant RNA MiniPrep kit (Zymo Research). The total RNA extracts were treated with RNase-free DNase (Invitrogen) and measured using Qubit (Invitrogen). RNA integrity was confirmed using RNA screen tape (TapeStation 2200, Agilent Technologies). Libraries were prepared from total RNA using a TruSeq stranded total RNA sample preparation kit, the Ribo-Zero plant kit (catalog no. RS-122-2401; Illumina). The final size and concentration of each library was verified using Qubit and D1000 screen tape (TapeStation 2200). Sequencing was performed by Macrogen Inc., South Korea with HiSeq 2500 using a TruSeq SBS kit version 4 (Illumina) with 151 cycles of paired-end reads by multiplexing 24 samples per lane. The reads were assembled and the genomes were annotated using CLC Genomics Workbench version 6.5 (CLC bio) and Geneious version 8.1.7 (Biomatters) (5). Further alignment was done by MAFFT (6).

FTA card sample 66AL yielded 12,661,700 reads and, after trimming, 12,518,915 remained. *De novo* assembly generated 859 contigs and 10,138 reads mapped to the contig of interest with a coverage of 135×. The final complete genome sequence length was 10,781 nucleotides (nt) containing the 5′ (96 nt) and 3′ (218 nt) untranslated regions. The new SPVG sequence coded for 10 proteins, as occurs with other potyviruses (7). A BLAST-based pairwise sequence comparison (8) revealed an isolate that most resembled South Korean isolates HG167 and United States isolate GWB-G. The pairwise nucleotide sequence identity between these two isolates and 66AL was 83.0%, which is well within the species demarcation limit of 76% for complete *Potyvirus* genomes (9, 10). The closest pairwise identities were therefore 83.0% to KM014814 (HG167) from South Korea, 83.0% to JN613805 (GWB-G) from the United States, and 82.9% to JQ824374 from Argentina. Since no SPVG was detected in any Australian samples also collected in 2015, there is a need for further sampling to establish whether SPVG has spread to Australia from nearby Southeast Asian countries. Comparison of any Australian SPVG genomic sequences found with ones from neighboring countries would then be required.

### Accession number(s).

The sequence was deposited in GenBank under the accession number KX279878 (66AL).
